# A Farm to Fork Risk Assessment for the Use of Wastewater in Agriculture in Accra, Ghana

**DOI:** 10.1371/journal.pone.0142346

**Published:** 2015-11-10

**Authors:** Prince Antwi-Agyei, Sandy Cairncross, Anne Peasey, Vivien Price, Jane Bruce, Kelly Baker, Christine Moe, Joseph Ampofo, George Armah, Jeroen Ensink

**Affiliations:** 1 Environmental Health Group, Department of Disease Control, Faculty of Infectious and Tropical Diseases, London School of Hygiene and Tropical Medicine, Keppel street, WC1E 7HT, London, United Kingdom; 2 Department of Epidemiology and Public Health, University College of London, 1-19 Torrington Place, London, United Kingdom; 3 London School of Hygiene and Tropical Medicine, Keppel Street, London, United Kingdom; 4 Faculty of Infectious and Tropical Diseases, London School of Hygiene and Tropical Medicine, Keppel street, London, United Kingdom; 5 Center for Global Safe Water, Emory University, Atlanta, Georgia, United States of America; 6 Water Research Institute (WRI), Council for Scientific and Industrial Research (CSIR), Accra, Ghana; 7 Noguchi Memorial Institute for Medical Research, College of Health Sciences, University of Ghana – Legon, Accra, Ghana; U. S. Salinity Lab, UNITED STATES

## Abstract

The need to minimise consumer risk, especially for food that can be consumed uncooked, is a continuing public health concern, particularly in places where safe sanitation and hygienic practices are absent. The use of wastewater in agriculture has been associated with disease risks, though its relative significance in disease transmission remains unclear. This study aimed at identifying key risk factors for produce contamination at different entry points of the food chain. Over 500 produce and ready-to-eat salad samples were collected from fields, markets, and kitchens during the dry and wet seasons in Accra, Ghana, and over 300 soil and irrigation water samples were collected. All samples were analysed for *E*. *coli*, human adenovirus and norovirus using standard microbiological procedures, and real time RT-PCR. Finally, critical exposures associated with microbial quality of produce were assessed through observations and interviews. The study found that over 80% of produce samples were contaminated with *E*. *coli*, with median concentrations ranging from 0.64 to 3.84 Log *E*. *coli/*g produce. Prepared salad from street food vendors was found to be the most contaminated (4.23 Log *E*. *coli/*g), and that consumption of salad exceeded acceptable health limits. Key risk factors identified for produce contamination were irrigation water and soil at the farm level. Storage duration and temperature of produce had a significant influence on the quality of produce sold at markets, while observations revealed that the washed water used to rinse produce before sale was dirty. The source of produce and operating with a hygiene permit were found to influence salad microbial quality at kitchens. This study argues for a need to manage produce risk factors at all domains along the food chain, though it would be more effective to prioritise at markets and kitchens due to cost, ease of implementation and public health significance.

## Introduction

Although the full extent of disease burden attributable to food-borne diseases is largely unknown, food hygiene and food safety are major public health concerns. In 2005, for example, the World Health Organisation (WHO) attributed 1.8 million diarrhoea-related deaths largely to contaminated food and drinking water [1]. In the United States alone, an estimated 9.4 million episodes of food-borne illness, with 55,961 hospitalizations and 1,351 deaths are recorded every year [2]. Food-borne diseases result not only from consuming food contaminated with pathogens such as bacteria, viruses and parasites, but also chemicals or bio-toxins [2, 3]. The most often reported microbial agents related to food-borne diseases are *Salmonella* spp, norovirus, *E*. *coli*, *Clostridium perfringens* and *Campylobacter* spp [1, 2].

The risk factors for produce contamination are diverse, and may include environmental, animal and human sources. The use of urban wastewater for irrigation, post-harvest practices including market handling, and poor hygienic practices at kitchens have all been linked to produce contamination and disease outbreaks [4]. Although the health risks arising from urban wastewater use in agriculture are well documented, how consumer risk changes from field, to market, and to household are unclear and poorly documented. In most past studies, there tends to be a focus on disease risks analysis at the farm domain with very little at the market and kitchen domains. This lack of systematic assessment of food hygiene and safety along the complete food chain was the main thrust for the development of the Hazard Analysis and Critical Control Points (HACCP) in the food industry [5]. The HACCP helps identify critical pathways along the food chain where interventions could be prioritised; hence, specific risk-based targets can be developed to control hazards at the different steps in the food production chain [5]. This study adopted a HACCP approach to identify key risk factors associated with the microbial quality of produce from farm to fork.

## Materials and Methods

### Study area and site selection

The study was conducted in Accra, the capital city of Ghana with a population of 1.9 million [6]. Seven major agricultural sites were identified in the city where poor-quality water was used for the cultivation of salad vegetables, including lettuce, spring onions, cabbage, and local vegetables. Most crops are irrigated through the use of watering cans. Farmers sell their produce mainly to market vendors, but also directly to restaurants and street food vendors. Markets in Accra are classified into five types: central markets, neighbourhood markets, night markets, specialist markets, and privately managed markets [7]. The central markets serve as the largest platforms for vegetable sales, attracting traders from both within and outside Ghana. In Accra, the street food sector constitutes one of the biggest informal categories within the food industry. A popular category of street food vendor in Accra is the “check-check” seller. These are vendors who mostly sell cooked, or fried rice with salad (fast-food). Salads are normally prepared from lettuce, cabbage, carrots or spring onions, and can be mixed with or, without salad cream.

The three largest wastewater irrigated sites in Accra were selected for this study—Korle Bu, Dzorwulu and Marine Drive. Only farmers with at least one bed of ready-to-harvest lettuce at the time of study were included. Farmers were randomly selected using their farm beds/plot as identification. Three central markets (Makola, Agbobloshie and Kaneshie) were also included in the study. Vendors who were thought to sell both cabbage and lettuce were included in the study, and were randomly selected using their market stalls as identification. ‘Check-check’ vendors were recruited from a list of food vendors previously identified by a transect walk in two neighbourhoods (Old Fadama and Alajo) in Accra. Restaurants (including hotels) in Accra where salad was served to the public were also included in the study during the rainy season, and were selected on the basis of their “star” rating, location and popularity from the database of restaurants and hotels from the Ghana Food and Drugs Authority (FDA).

### Data Collection

#### Sample collection and analysis

Lettuce, soil and irrigation water samples were collected from wastewater irrigated fields, while lettuce and cabbage were collected from local markets. Sample collection at farms and markets was done between 7:00 hrs and 10:00 hrs and between 18:00 hrs and 21:00 hrs at street vending sites, all at peak working periods. Ready-to-eat salad samples from restaurants were collected between 10:00 hrs and 15:00 hrs. All samples were collected from September to December 2012 in the dry season, and from July to August 2013 in the rainy season.

At farms and markets, farmers and vendors were asked to place produce directly into plastic sampling bags (Whirl-Pak, USA) after they had cut off any roots to prevent unrelated contamination. The temperature of produce at markets was taken just before sample collection using a hand-held meter (ETI 226–010 ThermaLite, ETI Ltd, UK). For prepared food, vendors were asked to place the food sample into the opened sampling bag using whatever means (e.g. hands, utensils) but a note was made on how the food was handled. The presence of flies, the distance to open drains, refuse, and defaecation areas were also recorded, while observations were made on how the produce was displayed during sample collection. All collected samples were placed in an ice-box, and transported to the laboratory within 2 hours of collection for immediate processing, or stored in a refrigerator at 4°C until processing. At the laboratory 500 ml of sterile PBS (phosphate buffered saline, pH 7.2) was added to the bags, which were then vigorously shaken, and the surface of each piece of produce gently massaged through the bag before being processed, and analysed for *E*. *coli*, human adenovirus and norovirus genomes I and II. A 10 g of ready-to-eat salad sample was measured into a sterile tube, vortexed and shaken vigorously at room temperature before the supernatant was processed for the *E*. *coli*, norovirus and adenovirus assays. For farm soil, 10 g of the sample was measured into a sterile tube and 20 ml of sterile PBS added to it before 10 ml of supernatant was used for the assays.

All samples were processed using the membrane filtration technique with BBL MI agar (Beckton Dickinson, Sparks, USA) to determine the prevalence and concentrations of *E*. *coli* [8]. Serial dilution ranges were pre-optimized to ensure that ranges allowed enumeration of roughly 95% of samples, per sample type. RNA was extracted using the Qiagen Viral extraction kit (Qiagen, Venlo, Netherlands), and DNA using the MPBio FastDNA kit for Soil (MP Biomedicals, Santa Ana, USA). Virus presence/absence and inhibition in water, soil and produce/prepared salad was determined using Quantifast Pathogen IC Real Time—Polymerase Chain Reaction (RT-PCR) and PCR kits. Norovirus GI and GII and adenovirus concentrations were determined using Qiagen OneStep kits [9].

#### Observations

Site observations were conducted in both seasons using structured observation guide, while participants’ observation was done in the dry season. Each farmer at wastewater irrigated fields and each vendor at markets was observed for one 3 hours session from 7:00 hrs to 10:00 hrs, whereas each street food vendor was observed from 18:00 hrs to 21:00 hrs. Farmers were observed during their farming activities including method of irrigation, application of poultry manure, and harvesting of produce. Market and street food vendors were observed on where and how they displayed and stored their produce, and any methods of treating produce/salad. In addition, general sanitation, including refuse, open drains, visible faeces, defecation areas as well as the presence of flies were observed. Participants were told that the observations were aimed at learning more about their general activities at farms, markets and street food vending sites, and not specifically to document critical health risk behaviours.

#### Questionnaire

At the end of each participant observation, a standardised questionnaire was verbally administered to farmers, market salespersons, street food vendors, as well as chefs at restaurants. Questionnaires explored the sources, and the methods of displaying produce at markets and ready-to-eat salad at kitchens. It also covered where vendors sold and how produce was stored. At kitchens, the method of treating salad leaves as well as when the salad was prepared were recorded. In addition, the personal characteristics of participants including age, sex, religion, occupation and education were recorded.

### Sample size

Sample size for produce was determined based on 80% power and 5% significance level to detect a 5% to 10% difference in faecal coliform concentration levels between produce at farms and markets [10]. This resulted in a sample size of 80 produce samples each from farms and markets during each of the dry and wet seasons. The number of soil and irrigation water samples collected at farms was assumed to correspond to the produce samples collected at farms during each season. Thirty samples of ready-to-eat salad were collected from 30 fast-food sellers (in each season) and 20 samples from chefs from 20 hotels and restaurants.

### Data Analysis

All data were analysed using STATA 12 (StataCorp LP, College Station, USA). All samples with undetectable concentrations were multiplied by 0.5, the lower limit of detection for *E*. *coli* dilutions, or per standard curve in molecular virology analysis. Distributions of *E*. *coli* concentrations in environmental and food samples were tested for normality using the Shapiro-Wilk test. Concentrations were log transformed for calculations of means, standard deviations, and 95% confidence intervals (CI). The Mann-Whitney test was used to test for the difference in median concentrations of *E*. *coli* of street vended salad and irrigation water between seasons, while the Kruskal-Wallis test was used to compare the median concentrations of produce samples among the different domains. Apart from street-vended salad, two sample t-tests were used to compare the mean *E*. *coli* concentrations of produce at farms, markets and restaurants between the dry and rainy seasons as their distributions were normal after log transformation. Linear and logistic multiple regression models were used to assess risk factors for produce quality. Using a forward stepwise-regression approach, only risk factors that were significantly associated with produce quality at 20% were included in the multiple regression model [11]. Prior information, significance test and the size of effect-measure modification were used to determine the inclusion of interaction terms in the model. Multicollinearity was assessed using the variance inflation factor [12]. Statistically significant associations in the multivariable analysis were measured at 5% significance level using the likelihood ratio test.

Microbial concentrations in irrigation water were reclassified as ≤ 3 Log *E*. *coli*/100 ml and > 3 Log *E*. *coli*/100 ml following the (old) 1989 WHO water quality standard set for wastewater use [13]. Concentrations in salad produce were also regrouped as ≤ 3 Log *E*. *coli*/g and > 3 Log *E*. *coli*/g [14] or ≤ 2 Log *E*. *coli*/g and > 2 Log *E*. *coli*/g which define guidelines limits considered as microbiologically satisfactory for consumption [15, 16]. The proportion of produce and prepared salad with *E*. *coli* concentrations that met these guideline limits were then noted.

### Quantitative Microbial Risk Assessment (QMRA)

In order to determine whether the consumption of wastewater irrigated produce met health standards, a quantitative microbial risk assessment (QMRA) model developed for the WHO guidelines for safe use of wastewater in agriculture was used [17]. The model uses the Karavarsamis-Hamilton method [18], together with the norovirus dose-response model developed by Teunis et al. [19]. A maximum tolerable additional disease burden of 10^-6^ disability-adjusted life years (DALYs) loss per person per year (pppy) as used in the WHO guidelines was adopted, which equates to a maximum permissible norovirus (NV) infection risk of 1.4 x 10^-3^ pppy.

The frequency and quantity of salad consumption were determined from questionnaire-based consumer surveys and laboratory experiments [20]. The amount of salad consumed on a daily basis at home was estimated using the average weight of lettuce bought at markets, and the number of lettuce used to prepare a salad meal for a family of 4 [20]. The quantity of salad consumed at street food vendor level was based on a national consumer survey by the International Water Management Institute in Ghana [21].

Five different consumption exposure, or pathway models were used to estimate the dose of norovirus ingested and subsequently, the risk of infection. The irrigation water model used the quality of irrigation water to estimate pathogen dose ingested based on the amount of wastewater left on produce after irrigation. All other models (farm produce model, market produce model, restaurant salad model and street food model) used direct *E*. *coli* concentrations on produce, or in prepared salad to estimate the dose ingested. A maximum pathogen reduction of 2 Log units arising from produce washing or disinfection was assumed for farm and market produce models [22]. No pathogen reduction was assumed for prepared salad models.

### Ethical Considerations

Ethical approval was received from the London School of Hygiene and Tropical Medicine (reference number—6236) and from the Noguchi Memorial Institute of Medical Research, Accra, Ghana (Reference number—DF22). The study was explained to and agreed by local leaders, and written informed consent was obtained from each individual study participant and counter-signed and dated by researchers. All participants in this study were adults and were assured of confidentiality and security of the information they provide. Participants could only be identified by the use of alphanumeric symbols. The London School of Hygiene and Tropical Medicine granted permission for fieldwork. Access to hotels and restaurants was granted by the Ghana Food and Drugs Authority (FDA), while local government leaders (Assemblymen) approved of access to street food vendors in their communities. Access to markets and urban agriculture fields were also granted by the Secretaries and Market Queens of the market associations and the leaders of the farmer associations respectively. The field work did not involve any endangered or protective species.

## Results

### Microbiological quality of produce

**Fig 1:**
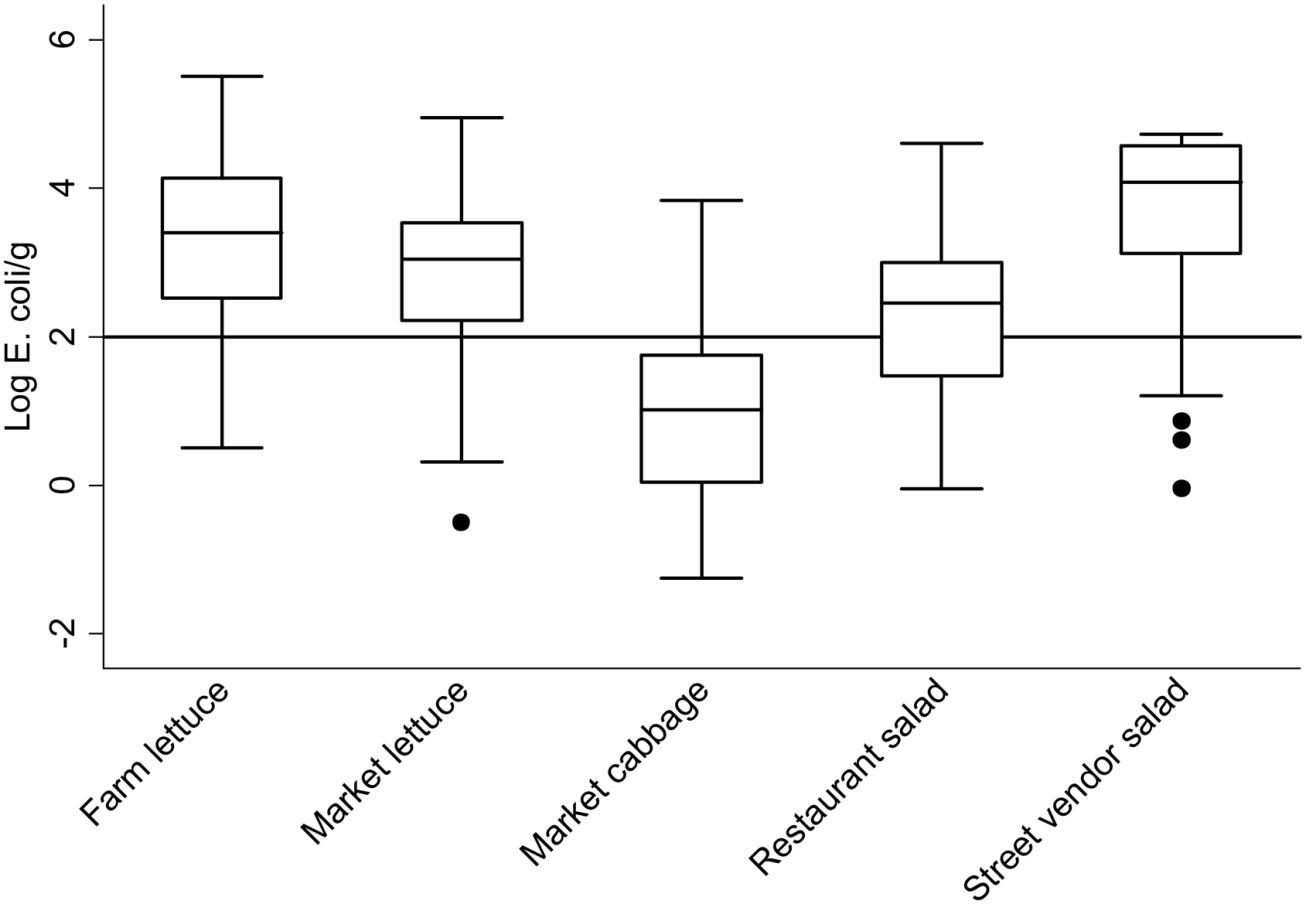
*E*. *coli* concentrations in raw produce and ready-to-eat salad at different entry points along the food chain. Solid horizontal line: limit of E. coli concentration classified as microbiologically satisfactory for consumption (% exceeding 2 Log E. coli/g—street vended salad, 90% (N = 59), farm lettuce, 88% (N = 159), market lettuce, 80% (N = 134), restaurants salad, 60% (N = 20), market cabbage, 18% (N = 129)). P-value calculated using Kruskal-Wallis test.

A total of 422 produce samples were collected, 159 from wastewater irrigated fields and 263 from markets (134 lettuce & 129 cabbages), and a further 79 samples of ready-to-eat salads (59 from street food vendors and 20 from restaurants). Ready-to-eat salad from street food vendors was found to be the most contaminated, with 98% of all collected samples positive for *E*. *coli*, followed by market lettuce (97%), farm lettuce, (96%), market cabbage, (89%) and restaurants salads (80%). Overall, street salad was found to have the highest concentrations of *E*. *coli* (4.1 Log *E*. *coli*/g) among all produce and prepared salad samples (Fig 1). Farm lettuce was found to contain significantly higher levels of contamination than market lettuce for the combined seasons (3.3 vs 2.9 Log *E*. *coli*/g, p < 0.001). The concentrations of *E*. *coli* on farm lettuce during the dry season were found to be higher than during the rainy season (Table 1), in contrast to lettuce and cabbage at markets, which were found to contain higher concentrations during the rainy season (Table 2).

**Table 1:** Risk factors for *E*. *coli* contamination of produce (lettuce) at farms.

Exposure	N	Mean (Log *E*. *coli*/g)	95% CI*	P—value**
Proximity to open drain				
≤ 3m	7	3.55	2.04, 5.05	0.12
> 3m	73	2.79	2.51, 3.07	
Irrigation water proximity to trash/refuse				
≤ 3m	59	2.92	2.57, 3.28	0.43
> 3m	21	2.67	2.29, 3.06	
Source of irrigation water				
Drain water	36	3.48	3.13, 3.83	< 0.001
Dug-out/pond	41	2.40	2.03, 2.77	
Piped water	3	1.61	-0.63, 3.84	
Irrigation water quality				
≤ 3.0 Log *E*. *coli*/100ml	24	2.52	1.98, 3.07	< 0.001
> 3.0 Log *E*. *coli*/100ml	130	3.46	3.27, 3.65	
When produce last irrigated				
≤ 2 days	45	2.97	2.66, 3.28	0.35
> 2 days	35	2.71	2.20, 3.22	
Soil with manure				
Yes	48	3.01	2.70, 3.32	0.19
No	32	2.63	2.11, 3.16	
Soil quality				
≤ 2.3 Log *E*. *coli*/g	81	3.00	2.72, 3.29	< 0.001
> 2.3 Log *E*. *coli/*g	76	3.64	3.41, 3.85	
Season				
Dry season	79	3.76	3.57, 3.95	< 0.001
Rainy season	80	2.86	2.58, 3.13	

*95% CI = 95% confidence interval.

**p-value calculated using t-test or ANOVA

**Table 2:** Risk factors for *E*. *coli* contamination of salad produce at markets.

	Lettuce				Cabbage			
Exposure	N	Mean (Log *E*. *coli*/g)	95% CI*	P—value	N	Mean (Log *E*. *coli*/g)	95% CI	P—value**
Season								
Dry season	54	2.53	2.27, 2.78	< 0.001	49	0.75	0.40, 1.10	0.06
Rainy season	80	3.12	2.92, 3.31		80	1.15	0.89, 1.40	
Type of market								
Main market (under roofing)	44	3.02	2.75, 3.28	0.28	36	1.07	0.68, 1.47	0.60
Open-air/street market	36	3.23	2.93, 3.54		44	1.21	0.86, 1.56	
Display of produce								
On ground (using mats)	15	3.21	2.75, 3.67	0.59	19	1.06	2.75, 3.67	0.92
> 1m above ground	41	3.02	2.73, 3.30		34	1.15	2.73, 3.30	
< 1m above ground	24	3.23	2.84, 3.61		27	1.20	2.84, 3.61	
Vending site concreted								
Yes	69	3.12	2.90, 3.34	0.87	71	1.20	0.92, 1.48	0.24
No	11	3.08	2.55, 3.59		9	0.72	0.11, 1.32	
Produce exposed to sunlight								
Yes	13	3.14	2.68, 3.61	0.90	21	1.16	0.56, 1.75	0.97
No	67	3.11	2.89, 3.33		59	1.15	0.86, 1.43	
Produce covered or not								
Yes	9	2.70	2.02, 3.37	0.13	4	1.33	0.43, 2.23	0.75
No	71	3.17	2.96, 3.38		76	1.14	0.87, 1.41	
Produce storage temperature								
≤ 25°C	23	3.24	2.83, 3.64	0.45	31	1.52	1.15, 1.90	0.02
> 25°C	57	3.01	2.83, 3.30		49	0.91	0.57, 1.25	
Produce storage time/hr	80	0.028	0.0088, 0.048	0.05	80	0.0021	-0.009, 0.013	0.69

SD° = standard deviation.

*95% CI = 95% confidence interval.

**p-value calculated using t-test or ANOVA

None of the produce samples from farms and markets were found to be positive for norovirus GI and GII, while 9% (N = 57) of farm produce, and 7% (N = 85) of market produce samples were positive for adenovirus. Mean concentrations of adenovirus in farm and market produce that tested positive for the virus were 8.1 x 10^3^ and 1.9 x 10^4^ gene copies/produce, respectively. No street vended salad sample was found positive for either one of the viruses.

### Key exposures and practices associated with produce quality

The use of poultry manure as soil fertilizer was common and was higher in the dry season than in the rainy season (99% vs. 60%). The study also found that open defecation was common among farmers (73%), though the practice normally occurred away from the main farming areas. Although 68% of market vendors reportedly washed their vegetables (lettuce and carrots) before sales, observation of vendors’ washing practices at markets showed that washed water for produce was used without changing it for an average of 22 minutes, and the washed water was always dirty. At markets, at least 80% of produce were sold within 24 hours, but in some cases could be stored for 48 hours for lettuce, and 84 hours for cabbage before sale. At the street food sites, vendors used either public toilets (73%), or market toilets (27%). Generally, environmental sanitation at most street food sites was poor, with 87% of the sites without concrete or cement floors (Table 3). The 3-hour observations revealed that 33% of street food vendors had their salads uncovered at the time of sampling, and that salad could remain uncovered for an average time close to 100 minutes. Four of the six vendors who were observed to prepare salad at their vending sites, did not wash their hands before salad preparation. At vending sites, produce was stored for an average time of 10 hours before being used or sold.

**Table 3:** Risk factors for *E*. *coli* contamination of ready-to-eat salad at street vending sites.

Exposure	N	Median (Log *E*. *coli*/g)	IQR*	P—value**
Season				
Dry season	29	4.23	3.60, 4.60	0.06
Rainy season	30	3.93	3.13, 4.57	
Proximity to open drain or refuse				
< 3m	23	3.95	2.98, 4.02	0.68
> 3m	7	3.13	2.42, 4.19	
Covered at time of sampling				
Yes	20	4.03	2.83, 4.32	0.61
No	10	3.63	2.92, 4.28	
Vending site concreted				
Yes	4	3.47	3.02, 3.87	0.39
No	26	4.05	2.76, 4.32	
Placement of salad in bag				
Plastic bag	3	4.32	4.31, 4.32	0.14
Spatula/spoon	21	3.37	2.76, 4.25	
Hands	6	4.12	2.92, 4.60	
Salad treatment method				
Salty water	17	3.82	3.11, 4.32	0.15
Vinegar	5	3.91	2.76, 4.15	
Salty water & vinegar	2	1.09	0.79, 1.38	
Water only	6	4.26	3.95, 4.32	
Where produce stored (n = 24)				
At home	3	4.58	4.21, 4.60	0.75
Vending site	11	4.01	3.60, 4.60	
Use immediately	10	4.31	2.62, 4.60	
How produce stored (n = 24)				
On a mat laid on ground	6	4.24	3.58, 4.60	0.26
In a box or container	11	4.60	3.60, 4.60	
Other	7	3.83	1.20, 4.38	
Where salad often prepared (n = 24)				
At home	5	4.58	4.23, 4.60	0.47
Vending site	19	4.08	3.58, 4.60	

*IQR = interquartile range

**p-value calculated using Mann-Whitney test or Krystal-Wallis test

### Risk factors for produce microbial quality

**Fig 2:**
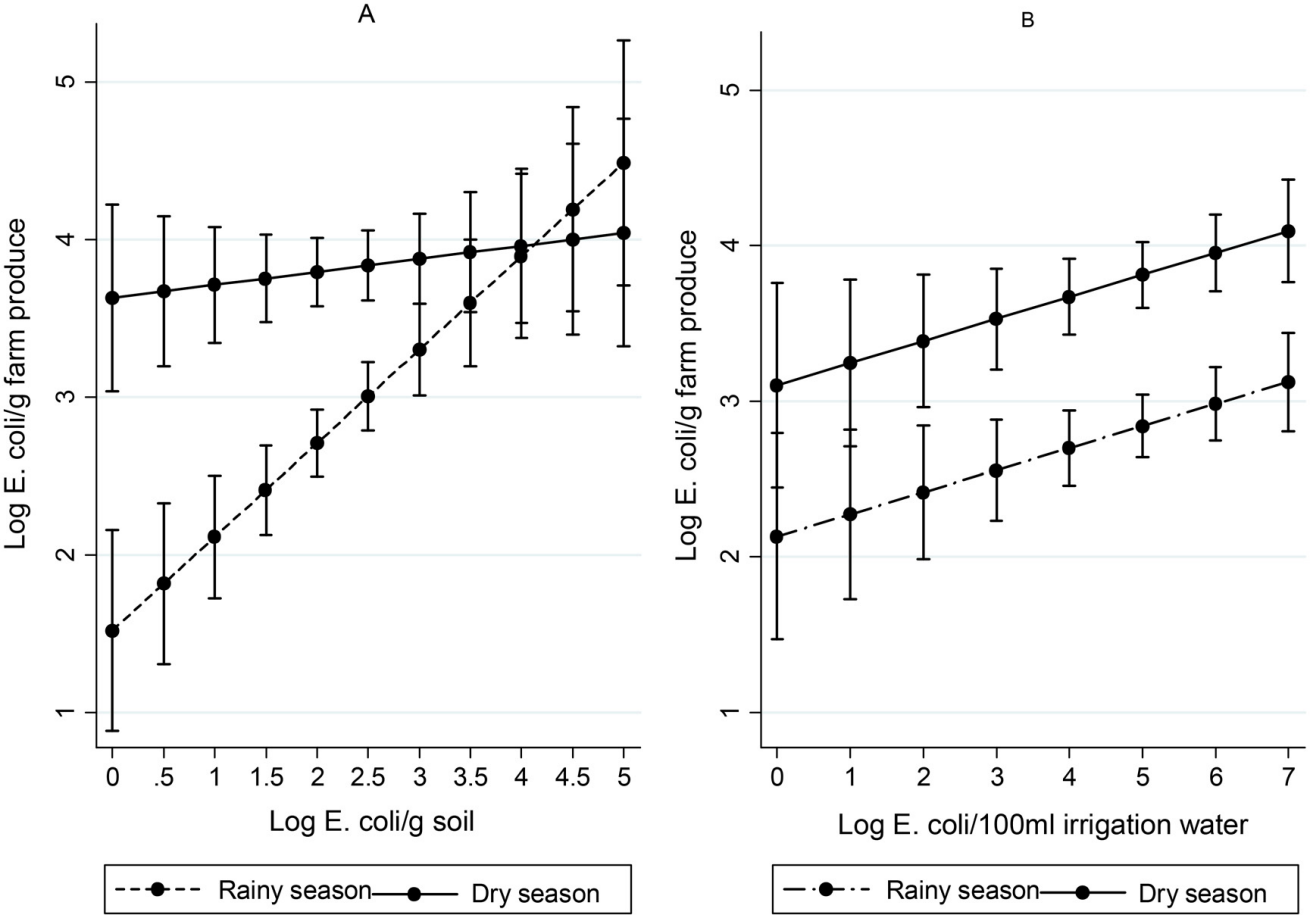
Effect of soil (A) and irrigation water (B) on farm produce quality after adjusting for seasonality. Seasonality and soil interaction p = 0.004, 95% CI = -0.85, -0.17. Soil effect on produce contamination (point estimate for unit increase = 0.60 Log E. coli/g produce, 95% CI = 0.32, 0.87, p < 0.001). Effect of irrigation water on produce contamination (point estimate for unit increase = 0.14 Log E. coli/g produce, 95% CI = 0.02, 0.27, p = 0.027). Error bars = 95% confidence intervals (CI).

The concentrations of *E*. *coli* found on farm produce increased with increased levels of *E*. *coli* found in soil or irrigation water (Fig 2). Seasonality modified the association between farm soil and farm produce quality with lower concentrations of *E*. *coli* found in the dry season as compared to the rainy season, with a 0.05 Log *E*. *coli*/g and 0.70 Log *E*. *coli*/g increase in produce contamination found per unit (Log *E*. *coli/g*) increase in soil contamination for the dry and rainy season respectively. In contrast, the effect of irrigation water quality on produce quality was found to be higher in the dry season as compared to the rainy season with a 0.20 Log *E*. *coli*/g and 0.06 Log *E*. *coli*/g increase in produce contamination per unit (Log *E*. *coli/*100 ml) increase of *E*. *coli* in irrigation water. However, the association between irrigation water and farm produce quality and the modification by seasonality was found to be non-significant (p = 0.19). The time of application of irrigation water, or poultry manure before sampling was found not to play any significant role on the concentration of *E*. *coli* found on farm produce (Table 1).

No environmental exposures were identified at the markets that influenced the concentrations of *E*. *coli* found on market produce. However, every 1 hour increase in the storage time of lettuce was significantly associated with a 0.03 Log *E*. *coli*/g increased levels of produce quality (p = 0.05). On average, lettuce was stored for 10 hours at markets but for a maximum of 48 hours before sales. During the rainy season, a 1°C increase in the storage temperature of cabbage resulted in a reduction of 0.28 Log *E*. *coli*/g in the produce quality.

The method reported to decontaminate salad had a non-significant impact on the quality of street salad (Table 3). At hotels and restaurants, those who operated with a valid hygiene permit had on average 1.53 Log *E*. *coli*/g less contamination of their salad than those who had no valid hygiene permit (Table 4). The source of raw produce was also associated with a borderline significant difference in the average *E*. *coli* levels in salad sold at restaurants with the highest contamination or impact resulting from produce bought directly from farm gates (Table 4).

**Table 4:** Risk factors for *E*. *coli* contamination of ready-to-eat salad at restaurants.

**Exposure**	**N = 20**	**Mean (Log *E*. *coli*/g)**	**95% CI**	**P**_**1**_ **–value**
*Univariable Analysis*				
Kitchen type				
Hotel	10	1.79	0.98, 2.59	0.22
Restaurant	10	2.56	1.43, 3.70	
Covered or not				
Covered	11	2.17	1.11, 3.23	0.99
Not covered	9	2.17	1.23, 3.12	
Hygiene Permit				
Yes	17	1.94	1.23, 2.65	0.08
No	3	3.48	2.43, 4.54	
When prepared				
Freshly prepared	11	1.88	0.92, 2.85	0.32
Already prepared	9	2.53	1.50, 3.55	
Source of produce				
Farm gate	4	3.24	1.18, 5.31	0.06
Wholesale market	7	1.14	-0.03, 2.32	
3rd-party supplier	7	2.39	1.27, 3.51	
Supermarket	2	2.88	-1.02, 6.79	
Placing of salad into sampling bag				
Spatula	13	2.00	1.10, 2.90	0.76
Hands	4	2.41	-0.38, 5.21	
Hands with gloves/plastic bag	3	2.61	1.95, 3.27	
Salad treatment				
Vinegar	3	1.77	-2.15, 5.70	0.88
Salt water & vinegar	6	1.95	0.002, 3.90	
Water only	3	2.25	0.23, 4.28	
Others	8	2.46	1.37, 3.55	
Storage time/hr	20	-0.030	-0.05, -0.0043	0.02
***Multivariable Analysis*** *				
**Exposure**	**Obs**	**Change in mean (Log *E*. *coli*/g)**	**95% CI**	**P**_**2**_ **–value**
Hygiene Permit	20	1.53	-0.03, 3.10	0.05
Storage time/hr	20	-0.015	-0.04, 0.011	0.24
Source of produce (farm gate as baseline)	20			
Wholesale market	7	-2.08	-3.56, -0.60	0.01
3rd-party supplier	7	-0.90	-2.34, 0.55	0.20
Supermarket	2	-1.45	-3.52, 0.61	0.15

P_1_, p-value calculated using t-test and Anova

P_2_, p-value calculated using likelihood ratio test.

*After controlling for hygiene permit, source of produce and storage time of produce

### Produce quality and infection risk associated with salad consumption

On average, consumers of street food consumed 13 g of salad 4 times/week, while domestic consumers consumed 51 g of salad 2 times/week [20]. The median norovirus infection risk for the consumption of 10–51 g of lettuce salad for 2–4 days per week varied across the different exposure models and ranged between 2.6 x 10^-3^ and 0.32 pppy, and was highest with the street salad model (Table 5). The estimated infection risks from the irrigation water model, restaurant, and street salad models all exceeded the WHO guidelines. Only the risks from the consumption of produce of average contamination levels at farms and markets were marginally within the acceptable norovirus infection risks.

**Table 5:** Median norovirus infection risks from the consumption of 10–51 g of wastewater irrigated lettuce on 2–4 days per week estimated by 10,000 Karavarsamis- Hamilton MC simulations.

*E*. *coli* contamination	Median norovirus infection risk (pppy)	95-percentile norovirus infection risk (pppy)
Water model (*E*. *coli*/100 ml)		
3.63 x 10^5^ ^†^	0.142	0.198
1.48 x 10^7^ ^‡^	0.997	1.0
Produce/salad quality (*E*. *coli*/100 g)		
Farm produce model		
2.04 x 10^5^ ^†^	6.8 x 10^-3^	9.9 x 10^-3^
3.16 x 10^7^ ^‡^	0.650	0.785
Market produce model		
7.59 x 10^4^ ^†^	2.6 x 10^-3^	3.7 x 10^-3^
8.91 x 10^6^ ^‡^	0.258	0.354
Restaurant salad model*		
1.48 x 10^4^ ^†^	1.3 x 10^-2^	1.9 x 10^-2^
3.98 x 10^6^ ^‡^	0.964	0.992
Street salad model*		
4.57 x 10^5^ ^†^	0.323	0.430
5.37 x 10^6^ ^‡^	0.988	0.998

Assumptions: 0.1–1 norovirus per 10^5^ *E*. *coli*, disease/infection ratio 1:1.

^†^ Median irrigation water contamination or mean produce/salad contamination

^‡^Maximum *E*. *coli* contamination

*No pathogen reduction for prepared salad at restaurants and street kitchens

## Discussion

The results of the study showed that salad produce was faecally contaminated at all entry points of the food chain, with street salad being the most contaminated. Key risk factors identified included farm soil contamination, and the use of wastewater for irrigation. Others were poor food and environmental hygiene, produce storage time and temperature, and operating without a hygiene permit. Based on the WHO QMRA model, the consumption of salads in Accra exceeded permissible health risk standards [23].

### Produce quality from farm to fork

This study found street food salad to be the most faecally contaminated food, though concentrations of *E*. *coli* found at all sample points were high. The concentrations of faecal pathogens found on lettuce at farms in this study (3.31 Log *E*. *coli*/g) were lower than those found in previous studies in Ghana, ranging from 5.0 Log MPN/g to 9.0 Log MPN/g [24, 25], though most studies reported contamination in concentrations of total thermotolerant coliform (TTC), and not *E*. *coli* specifically [26]. However, the *E*. *coli* concentrations found on lettuce collected from agricultural fields in Accra were significantly higher than those in Pakistan [27] (>2,000 *E*. *coli*/g vs 1.9 *E*. *coli*/g produce), even though the irrigation water quality was found to be much better in Ghana (9.8 x 10^4^ vs 1.8 x 10^7^ *E*. *coli*/100 ml). This could most likely be explained by the type of irrigation water application, watering cans in this study, as compared to basin or furrow irrigation techniques, which minimize contact with wastewater; while the much higher temperatures and lower humidity in Pakistan could have promoted much more rapid die-off of *E*. *coli* on produce [27].

The microbial quality of produce is influenced by a variety of factors, which include: the type of vegetable, environmental conditions like temperature, humidity and exposure to sunlight, the type and the application of irrigation water, and post-harvest handling. Studies have shown that leafy vegetables with irregular surfaces tend to be more contaminated than smooth surface vegetables like cabbages [28, 29], and this could explain the higher levels of *E*. *coli* on market lettuce than cabbage in this study. A similar trend was also found by Amoah [24] in three cities (Accra, Kumasi and Tamale) in Ghana, where lettuce was found to harbour higher levels of faecal coliform than cabbage (1.1 x 10^7^/g vs. 3.3 x 10^6^/g).

The concentrations of *E*. *coli* in street food salad in this study (3.7 Log *E*. *coli/*g), although high, were lower than levels found in earlier studies in Accra, which were found to range between 5.1 and 6.4 Log cfu/g [30–34], though Mensah et al. [34] measured faecal coliforms. The higher levels of faecal contamination in those studies could be attributed to the fact that the salad were mixed with salad cream containing egg yolks, which has shown to be a good medium for microbial growth [30, 35]. Salad samples collected for this study were without salad cream. Most previous studies have also associated the contamination of street salad or raw produce to the sources of the produce, transportation practices, storage practices, method of salad preparation, and poor sanitation [30, 33, 36]. Similarly, the contamination of street food salad in this study could be associated with poor sanitation and hygiene practices at the vending sites. A study in Kumasi found higher levels of the TTC on produce (1.8 Log units/100 g) in salad from vendors with dirty sites and poor food handling practices [37]. The cut or sliced nature of the prepared salad also facilitated the growth of microorganisms, or increased their persistence, unlike raw produce which are intact [35]. The higher contamination of street food salad than salad from restaurants also confirms the findings of another study in Ghana, which found higher concentrations of TTC in salad sold by street vendors than those sold at cafeterias (5.4 Log cfu/100 g vs 3.8 Log cfu/100 g) [37].

The higher contamination of produce at farms than at markets emphasises the debate on the relative importance of post-harvest effect, including poor sanitation and market handling, on the quality of produce. Results from this study agree with earlier findings from Ghana [38], but contrast with the findings in Pakistan, where produce collected at a local market was found to have seven times higher levels of *E*. *coli* than produce from farm gates [27]. Apart from differences in irrigation methods, and environmental conditions that contributed to rapid pathogen die-off between the Ghana and Pakistan studies, the current study also seems to suggest that the differences might partly be influenced by seasonality, since farm produce was more contaminated than market produce only in the dry season and not in the rainy season. The design of the current study did not permit a direct correlation of farm-level contamination to produce contamination at markets, because produce at farms were not followed to markets to monitor the contamination levels. The geometric mean levels of *E*. *coli* found on market lettuce in this study (339 *E*. *coli*/g and 1,318 *E*. *coli*/g) were 20 times higher than levels found at markets in Faisalabad, Pakistan (14.3 *E*. *coli* /g) [27]. The contamination at Faisalabad was attributed to unsanitary market conditions and handling practices, including the method of washing produce.

The prevalence of human adenovirus (HAV) on farm produce was the first time viruses have been isolated in farm lettuce at the study sites. A recent study at the same sites analysed for human adenovirus and norovirus in wastewater, but not on farm produce [39]. Apart from norovirus, HAV is a known cause for food-borne infections, and its presence on farm produce could be attributed to the direct use of wastewater and animal manure for vegetable cultivation [40]. Moreover, viruses can survive on harvested produce and can remain infectious for several days up to a period of 5 weeks, even during storage [41, 42], and therefore could pose public health concerns. The study did not analyse samples for helminths, though earlier studies in Ghana have found helminth eggs on farm and market produce, and also in prepared salad sold at street food kitchens and also at cafeterias [10, 37].

### Health risks associated with produce quality

Despite the increasing recognition of international guidelines on food safety such as the Codex Alimentarius, there are still some variations on the acceptable level of microbial concentrations in ready-to-eat food at different countries. While the International Commission on Microbiological Specifications for Foods (ICMSF) recommends a limit of 1,000 *E*. *coli*/g produce, the United Kingdom has a threshold of < 20 *E*. *coli*/g as satisfactory for consumption, and ≥ 100 *E*. *coli*/g as unsatisfactory for ready-to-eat food (including fresh vegetables and mixed salad vegetables) at the point of sale, a standard also adopted by Canada, Australia, New Zeeland and Hong Kong [16]. In Ghana, the national microbiological reference value for ready-to-eat foods including salad is < 100 cfu/g, and is based on the 3-class attribute sampling plan (satisfactory, acceptable, and unsatisfactory) [15]. This is the minimum count of organisms per gram, or per ml, below which there would be no risk associated with safety of a food, or the maximum value beyond which a lot would be rejected. Based on the Ghana standards, only 11% and 20% of the lettuce collected from the wastewater fields, and local markets could be deemed safe, and 40% and 10% from restaurants and street food vendors respectively. Results from the WHO QMRA model also seem to suggest that, the use of wastewater, and post handling practices at markets and kitchens in Accra are unsafe since the estimated pathogen risks were higher than the recommended level of 1.4 x 10^-3^ pppy for norovirus. However, for a relaxed DALY loss of 10^-4^, as proposed by Mara [43], almost all the median annual norovirus risk arising from the use of average produce/salad contamination were within the norovirus acceptable limits (0.14 pppy), though all the worst case scenarios exceeded this limit. Although all exposure models in this study were approximations, the restaurant and street salad models represented the closest estimate of the risk to consumers since these models used fewer assumptions, and are also at the points of direct consumption. The irrigation water model is the least reliable, and should not be used in instances where direct concentrations of pathogens on produce, or in prepared salad are available. The main disadvantage of the irrigation water model is that it assumes that the use of wastewater is the only source of produce contamination, and does not account for other sources of contamination at farms, markets and kitchens, nor does it take into consideration die-off between field and fork, or produce washing procedures. Only the risks from the farm and market produce models were marginally within the acceptable risks, and this could be due to the inclusion of pathogen reduction measures prior to consumption in those models as described in the methods section. On the other hand, the risks from these models still exceeded the threshold limits if the highest level of produce contamination were considered.

All models were based on an indicator-pathogen ratios, and this is a serious limitation of the models, and can lead to spurious results due to the complexities in these relationships, and their poor correlation with actual pathogen concentrations. Moreover, there is inadequate evidence to support the widely used ratio of 1:10^5^ between bacteria indicator organisms and viruses, as the results from other studies seem to suggest that this ratio could result in overestimation of the risk. For example, a recent study [39] in Ghana found an average of one norovirus GII to 10^3.2^ *E*. *coli* from its quantifiable irrigation water samples, while a component of the current study [44] also found the ratio of means between norovirus and *E*. *coli* as 2.1 x 10^-2^ or 1:10^1.7^ from irrigation water samples analysed for both *E*. *coli* and norovirus, which are all larger than the common ratios used in recent publications. The presence of *E*. *coli* is an indicator of faecal pollution, but not necessarily a good indicator of disease risk, though signals the possible presence of other pathogens, and hence the need to intervene appropriately since “absolute zero risk” does not exist when assessing microbial risk in food [45]. In order to protect consumer health, a combination of produce washing and disinfection, which have been shown to reduce up to 3-Log unit of pathogens including norovirus is recommended; together with good agricultural practices [22, 46, 47]. Wastewater treatment and crop restriction are key risk reduction measures, but are rarely implemented in low and middle income countries.

### Risk factors, health protective measures and policy implications

Probably the most significant public health concern found in this study was the high levels of faecal contamination found in street food salads. This is of particular concern, as up to 800,000 people per day have been estimated to consume this food, and other salad related foods from food establishments in major cities in Ghana [21]. The results of the study did not identify specific risk factors for street vended salad, though the use of some cleaning methods seemed to have a protective effect. The time between salad preparation and consumption could be a potential risk factor, as studies have shown that though most sanitizing solutions are capable of reducing microbial concentrations following washing, epiphytic microorganisms can grow rapidly, reaching similar levels as before washing [48, 49]. A key recommendation for street food vendors, therefore, will be to prepare salad in small quantities based on customer inflow, in order to prevent contamination due to inadequate storage and inappropriate temperatures. Another recommendation would be to prevent the use of leftover salad mixing with freshly prepared salad which could be another source of cross contamination [30]. Salads sold at hotels and restaurants must be prepared upon customer request, or be refrigerated (below 5°C) until ready to be served. Generally, microbial growth is slowed down, or stopped at temperatures below 5°C or above 60°C, though some psychotrophic microbes (e.g. *Listeria monocytogenes*, *E*. *coli*) may still develop, or multiply below 5°C if storage time is too long [50–52].

The potential effect of farm soil, and irrigation water on salad contamination could not be assessed due to the limitation of the study design. Observations at farm sites showed that some street food vendors bought vegetables directly from farms, and sometimes washed their produce with irrigation water, a practice that has also been reported among other market vendors in other parts of Ghana [24]. Other possible sources of salad contamination could be the chopping board, cutting knives, and working surfaces used for food preparation at kitchens, especially if these devices were used for multiple purposes such as cutting of meat [53, 54]. Street food vendors’ practice of not covering salad properly in receptacles during sales could worsen the microbial load due to cross-contamination [35]. This study found lower levels of contamination when a hygiene certificate was in place suggesting that local authorities should require vendors to obtain one. In order for these to work, frequent hygiene inspection, and monitoring of food premises by food hygiene authorities should remain a key priority. This is particularly necessary since vendors’ knowledge, awareness, and attitudes on hygiene alone do not necessarily translate into good hygienic practices [55]. Aside from cooking of vegetables and thorough washing and disinfection, domestic consumers can also remove the outer parts of vegetables before salad preparation to reduce the potential risk of pathogens, since most pathogenic contaminations are exogenic [56].

Produce quality at market showed non-significant associations with hygiene and sanitation practices, which could be as a result of this study unable to measure other potential risk factors for produce quality at the markets. Irrespective of this limitation, two possible reasons could account for produce contamination at markets. At first, washing of produce, could introduce microbial contaminants if wash water was contaminated as was shown at markets in Portugal [57] and Pakistan [27]. A study in Bangladesh, found wash water used for fresh vegetables and fruits to have enteric bacteria concentrations ranging from 1.3 10^3^ to 2.0 10^7^ cfu/ml [58]. Vendors are advised to wash produce under running potable water, or use multiple batches of potable water in order to prevent produce recontamination, though this requires involvement of local authorities as many markets lack access to clean water. A second reason for produce contamination at market could be environmental contaminants, as observations showed that 90% of vending sites were made of concrete, and therefore reducing the risk of dust as a potential source of produce contamination [59]. This study could not assess the role of factors such as transportation practices along the distribution chain, and handling during storage which could contribute to produce contamination at markets.

At farm level, contaminated soil and wastewater were found to be the main risk factors for produce quality. The higher impact of soil contamination on farm produce quality in the rainy season than in the dry season could be due to the frequent splashes of soil on produce arising from rainfall, and has been suggested by others [24, 60]. The use of poultry manure did not show significant association with produce quality, though this could be due to the fact that data was only collected on reported manure use, within the last four weeks. Similar findings were reported during field trials in Accra [24, 38], where direct wastewater use was the major risk factor for produce contamination, with contaminated soil and poultry manure identified as other potential sources of contamination. In this field trial study, the use of manure increased the levels of faecal coliform on lettuce cultivated on average by tenfold, while the use of wastewater increased faecal coliform levels 0–100 fold. The high use of manure in this study was similar to those reported in previous studies (73% to 98%) in Accra and Kumasi, where manure was found to be highly contaminated with faecal coliforms ranging from 1.0 10^3^ to 1.0 10^8^/100 g [10, 38, 61]. Poultry manure is recommended only as soil amendment if it has been adequately dried, i.e. composted aerobically to levels between 60°C and 80°C, and for at least 15 days before application [51], something which was not reported to be practiced by farmers. Restrictions on the use of untreated wastewater, the adoption of crop restriction, together with drying of poultry manure remain the best ways to ensure food safety in Accra. However, these measures are difficult to implement in resource constrained settings as a result of high cost, lack of alternative sources of irrigation water, and farmers’ unwillingness to cultivate non-vegetables, or non-food crops for loss of profits. In countries where good quality irrigation water sources are unavailable in the short to medium term, interventions that require less restrictions and minimal financial investment from farmers are recommended, as farmers find these more attractive to adopt [62]. These interventions include agricultural practices that limit produce contact to sediments such as controlled fetching of irrigation water from sedimentation ponds, the use of watering cans fitted with caps or fabric filters to reduce splashes of contaminated soil on produce, and the use of simple filters [60, 62, 63]. These interventions, however, need to be tested widely to assess their implementation successes and challenges. Education on the proper use of manure, as well as local authorities’ collaboration and support to farmers to gain access to alternative fertilizers, and water sources such as wells are also significant measures that can be taken to mitigate exposure and health risks.

## Conclusions

The results of this study suggest that the use of untreated wastewater poses significant risks for produce contamination at the farm level, but its role in influencing consumer risks at markets and kitchens remains unclear. Though produce was contaminated at all entry points of the food chain, street food was found to be the most contaminated, and therefore the most critical domain because it is at the actual point of consumption, and also due to the large number of people who consume street food, and the fact that it poses the greatest risk to public health as was supported by the QMRA models. The study recommends field trials, or related studies, to establish the effect of wastewater irrigation on produce quality and hence consumer risks at markets and kitchens, and whether this effect is significant in terms of public health. It also recommends an assessment on the influence of produce washing practices at markets, and vendors’ preparation, handling and management of salad during sales, on the quality of salad produce. Lastly, further studies are needed to determine the presence and concentrations of pathogens to help improve the quality of QMRA estimates. The study concludes that, in as much as interventions at the source of production (farms) may result in significant positive public health impact, adequate hygienic practices including hygiene inspection, hygiene certification and improvements in general environmental and food hygiene practices at markets, but especially at points of consumption (food kitchens) are regarded as more effective in terms of cost, ease of implementation and above all public health significance. At farm sites, access to credit schemes and improved land security are recommended measures to encourage farmers to adopt risk reduction measures.

## Supporting Information

S1 Supporting Information  Appendix A. Irrigation water sample collection form
 Appendix B. Particulate (soil) sample collection form
 Appendix C. Raw produce (farm) sample collection form
 Appendix D. Raw produce (market) sample collection form
 Appendix E. Food (salad) sample collection form
 Appendix F. Questionnaire for farmers
 Appendix G. Questionnaire for market vendors
 Appendix H. Questionnaire for produce buyers (market)
 Appendix I. Questionnaire for street food vendors
 Appendix J. Questionnaire for street food consumers
 Appendix K. Observation guide for farm workers
 Appendix L. Observation guide for market vendors
 Appendix M. Observation guide for street food vendors
(PDF)
