# Supplementary material for: A Farm to Fork Risk Assessment for the Use of Wastewater in Agriculture in Accra, Ghana
Source: PLoS One. 2015 Nov 10;10(11):e0142346. doi: 10.1371/journal.pone.0142346 (PMC4640866; doi:10.1371/journal.pone.0142346)
Supplement: S1 Supporting Information — Appendix A. Irrigation water sample collection form Appendix B. Particulate (soil) sample collection form Appendix C. Raw produce (farm) sample collection form Appendix D. Raw produce (market) sample collection form Appendix E. Food (salad) sample collection form Appendix F. Questionnaire for farmers Appendix G. Questionnaire for market vendors Appendix H. Questionnaire for produce buyers (market) Appendix I. Questionnaire for street food vendors Appendix J. Questionnaire for street food consumers Appendix K. Observation guide for farm workers Appendix L. Observation guide for market vendors Appendix M. Observation guide for street food vendors (PDF) [file pone.0142346.s001.pdf]

S1 Supporting Information

Appendix A. Irrigation water sample collection form

Irrigation Water Sample Collection Form

Barcode/sample Id [ ]

Date [ ] / [ ] / [ ]

Time [ ] [ ]

1. GPS latitude [ ]
2. GPS longitude [ ]
3. Location ID: [ ]

Location Description [ ]

4. What is the source of irrigation water collected?

Drain Water ☐
Channelled Stream/River ☐
Dug-out/Pond ☐
Piped Water ☐
Shallow Well ☐
Tube/Deep Well ☐

5. If "Yes", check box:

Within 3m of trash? ☐
Within 3m of faeces? ☐
Within 30m of latrine or defaecation area? ☐

Field Notes: [ ]

6. Physiochemical Characteristics

Turbidity [ ]

Temperature [ ]

Electrical conductivity [ ]

7. At lab:

Starting Volume (L) [ ]

Lab notes: [ ]

Personal Information

8. Sex Male ( ) Female ( )
9. Age (yrs.) .[ ]
10. Religion Christian ( ) Moslem ( ) Traditional worshipper ( ) Other ( )
11. Educational background: No formal education ( ) Primary education ( ) JHS/MS ( ) SHS/A-level ( ) Polytechnic/University ( )

Date: **Time:**

Enumerator ID: **Location ID:**

Appendix B. Particulate (soil) sample collection form

Particulate (Soil) Sample Collection Form

Barcode/sample Id

Date __ / __ / __

Time __ __

1. GPS latitude
2. GPS longitude
3. Location ID:

Location Description

4. If "Yes", check box:

Exposed to sunlight? ☐
Within 3m of faeces? ☐
Ask farmer if soil is contaminated with poultry manure ☐

Field Notes:

5. At lab:

Weight (g)

Personal Information

6. Sex Male ( ) Female ( )
7. Age (yrs.) ...
8. Religion Christian ( ) Moslem ( ) Traditional worshipper ( ) Other ( )
9. Educational background: No formal education ( ) Primary education ( ) JHS/MS ( ) SHS/A-level ( ) Polytechnic/University ( )

Date: **Time:**
Enumerator ID: **Location ID:**

Appendix C. Raw produce (farm) sample collection form

Raw Produce (Farm) – Sample Collection Form

Barcode/sample Id ☐

Date ☐/☐/☐

Time ☐ ☐

1. GPS latitude ☐
2. GPS longitude ☐
3. Location ID: ☐
4. Select the source of raw produce
   Farm ☐
   Market ☐
   Home ☐
5. Tick the type of raw produce
   Lettuce ☐ Carrot ☐
   Cabbage ☐ Spring Onion ☐
6. Enter the number collected: ☐
7. Indicate when (in hours) irrigation was last done on produce before sampling: ☐
8. How produce placed in whirl-pak bag (select one):
   Plastic or paper wrap ☐
   Hands ☐
   Other ☐ Specify: ☐
9. Mark if the sample was taken
   Within 3m of faeces ☐
   Within 30m of latrine ☐
   With flies on food ☐
   Within 3m of sewage outfall Or open drain ☐
10. At lab:
   Weight (g) ☐ or Volume (mL): ☐
11. Mark how produce was covered
   Covered ☐
   Not covered ☐
12. Produced exposed to Sunlight:
   Yes ( ) No ( )

Notes: ☐

Personal Information

13. Sex Male ( ) Female ( )
14. Age (yrs.) . ☐
15. Religion Christian ( ) Moslem ( ) Traditional worshipper ( ) Other ( )
16. Educational background: No formal education ( ) Primary education ( ) JHS/MS ( ) SHS/A-level ( ) Polytechnic/University ( )

Date: **Time:**

Enumerator ID: **Location ID:**

Appendix D. Raw produce (market) sample collection form

Raw Produce (Market) - Sample Collection Form

Barcode/sample Id ☐

Date ☐ / ☐ / ☐

Time ☐ ☐

1. GPS latitude ☐
2. GPS longitude ☐
3. Location ID: ☐
4. Select the source of raw produce
   Farm ☐
   Market ☐
   Home ☐
5. Tick the type of raw produce
   Lettuce ☐
   Cabbage ☐
6. Enter the number collected: ☐
7. Type of market vendor : a) At main market (under shed) b) Open/street market (outside main market)
8. A). Indicate how long (hours) produce has been stored ☐
   B). Record produce storage temperature ☐
9. How produce placed in whirl-pak bag (select one):
   Plastic or paper wrap ☐
   Hands ☐
   Other ☐ Specify: ☐

10. Mark if the sample was taken
Within 3m of faeces ☐
Within 30m of latrine ☐
With flies on food ☐
Within 3m of sewage outfall Or open drain ☐

11. Mark how produce was covered
Covered ☐
Not covered ☐

12. Produced exposed to Sunlight:
Yes ( ) No ( )

13. How produce is displayed: a) On the ground b) >1m above ground c) <1m above ground d) Other

14. Is vending site concreted?
Yes ( ) No ( )

Notes: ☐

11. At lab:
Weight (g) ☐ or Volume (mL): ☐

Personal Information

16. Sex Male ( ) Female ( )
17. Age (yrs.) ☐
18. Religion Christian ( ) Moslem ( ) Traditional worshipper ( ) Other ( )
19. Educational background: No formal education ( ) Primary education ( ) JHS/MS ( ) SHS/A-level ( ) Polytechnic/University ( )

Appendix E. Food (salad) sample collection form

Food (Salad) Sample Collection Form

Barcode/sample Id [ ]

Date [ ] / [ ] / [ ]

Enumerator ID: [ ]

Time [ ] [ ]

1. GPS latitude [ ]
2. GPS longitude [ ]
3. Location ID: [ ]
4. Select the source of ready-to-eat food
   Street food ☐
   Hotel ☐
   Restaurant ☐
5. Select type of vendor
   Mobile ☐
   Non-mobile ☐
6. Indicate how long (days/hrs) produced has been stored before using for salad [ ]
7. Source of vegetables used to prepare salad: a) Farm gate b) Wholesale market c) Retail market d) Other (Specify): [ ]
8. How food was placed in whirl-pak bag (select one):
   Plastic or paper wrap ☐
   Spoon/spatula ☐
   Hands ☐
   Other ☐ Specify: [ ]
9. How salad was treated: a) Salt water b) Vinegar c) Salt Water & vinegar d) water e) other (specify) [ ]
10. Mark if the sample was taken
    Within 3m of faeces ☐
    Within 30m of latrine ☐
    With flies on food ☐
    Within 3m of sewage Outfall Or open drain ☐
    Within 3m of refuse ☐
11. At lab:
    Weight (g) [ ] or Volume (mL): [ ]
12. Mark how salad was covered
    Covered ☐
    Not covered ☐
13. Is vending site concreted?
    Yes ( ) No ( )
14. If hotel, class of hotel [ ]
15. Do you have hygiene permit to operate? Yes ( ) No ( )
16. If yes, from where? a) FDA b) AMA c) Other (specify)..

Notes [ ]

Personal Information

17. Sex Male ( ) Female ( )
18. Age (yrs.) . [ ]
19. Religion Christian ( ) Moslem ( ) Traditional worshipper ( ) Other ( )
20. Educational background: No formal education ( ) Primary education ( ) JHS/MS ( ) SHS/A-level ( ) Polytechnic/University ( )

Appendix F. Questionnaire for farmers

QUESTIONNAIRE FOR FARMERS.

A. Environmental Hygiene conditions

1. Where do you normally defaecate when at work on the farm?
   Public toilet ( ) toilet on the farm premises ( ) A neighbour's toilet ( )
   Open Defaecation ( )
2. What is your source of drinking water when working on the farm? (Tick all responses)
   Sachet water ( ) Bottled mineral water ( ) Piped water ( ) Water from home ( )

B. Food Hygiene practices

3. How many times do you eat when at work? Once ( ) 2 times ( ) 3 times ( )
   More than 3 times ( )
4. What is the source of the food you normally eat when at work?
   Food from home ( ) street food ( ) local restaurant food ( ) prepared food on farm ( ) Other ( )
5. Which of these do you do most?
   Eating at the farm ( ) Eating at the food vending site ( ) Eating at home ( ) Other ( )
6. Do you normally wash your hands before eating at the farm site?
   Yes ( ) No ( )
7. If Yes, what do you wash your hands with?
   Irrigation Water only (Specify)...................... Pipe water only ( ) Sachet Water only ( )
   Soap and water ( )

C. Health Risk Perceptions and Awareness

8. Are you aware of any health risks associated with farming practices using drain water?
   Yes ( ) No ( )
9. If yes, what kind of health risk do you know of?
   List all of them...
10. What do you do to protect yourself from these health risks?
    List them...

D. Socio-economic issues

11. Does the use of drain water/dug-out water for irrigation increase your income as compared to the use of piped water?
    Yes ( ) No ( ) Cannot tell ( )
12. Is farming the main source of your income? Yes ( ) No ( )
13. How much time do you spend on the farm?...........
14. If you have any other job, how much time do you spend at:
    Farm...................... Other job (s)............................
15. How many people depend on you? [ ]

E. Personal Information

16. Sex Male ( ) Female ( )
17. Age [ ]
18. Religion Christian ( ) Moslem ( ) Traditional worshipper ( ) Other ( )
19. Educational background: No formal education ( ) Primary education ( ) JHS/MS ( ) SHS/A-level ( ) Polytechnic/University ( )

Date: **Time:**
Enumerator ID: **Location ID:**

Appendix G. Questionnaire for market vendors

QUESTIONNAIRE FOR MARKET VENDORS

A. Produce Hygiene conditions

1. Where do you normally store your produce before selling them?
   At the market ( ) At home ( ) other ( ) specify
2. How do you normally store the following vegetables before selling them? (Indicate in the table below):

**Vegetables**	**How storage is done**
a) Lettuce	
b) Carrots	
c) Spring Onions	
d) Cabbage	

   In a sack ( ) In a basket ( ) On a table but covered ( )
   In a box ( ) Other ( ) specify..

3. Where do you normally display your vegetables for sale? (Indicate in the table below):

**Vegetables**	**How produce is displayed**
a) Lettuce	
b) Carrots	
c) Spring Onions	
d) Cabbage	

   Table top ( ) Basket/bowl ( ) material on ground ( ) bare ground ( )

4. Do you wash the vegetables before sales?
   Yes ( ) No ( )
5. If yes, what is the source of water for washing the vegetables?
   Piped water ( ) well water ( ) Tanker services ( ) Other (specify)...................
6. How much time do you spend on the following activities daily

**Activity**	**Time Spent**
a) Washing of lettuce	
b) Washing of carrot	
c) Cutting or removal of waste parts of cabbages	
d) Removal of waste parts of spring onions	

7. Are customers normally happy with the quality of vegetables sold at the market?
   Yes ( ) No ( )
8. What do customers normally complain of when at the market?
   Write complaints..
   ....

B. Environmental Hygiene conditions

9. Where do you normally defaecate when you are at the market?
   Public toilet ( ) Market toilet ( ) In a polythene bag ( ) Open Defaecation ( )

10. What is your source of drinking water when working at the market?
    Sachet water ( )   Bottled mineral water ( )   Piped water ( )   Water from home ( )
11. Are you normally satisfied with refuse collection and management at the market?
    Yes ( )   No ( )
12. If No, what are you not satisfied with?
    Write down reason (s)..
13. Are you generally satisfied with drainage management at the market?
    Yes ( )   No ( )
14. If No, what are you not satisfied with?
    Write down reason (s)..

C. Hand washing and food Hygiene practices

15. How many times do you eat when at the market?
    Once ( )   2 times ( )   3 times ( )   more than 3 times ( )
16. What is the source of the food you normally eat when at the Market?
    Food from home ( )   street food ( )   local restaurant food ( )   Other ( ) specify.......................................
17. Do you normally wash your hands when eating at the market?
    Yes ( )   No ( )
18. If Yes, what do you wash your hands with?
    Only water ( )   Soap and water ( )

D. Health Risk Awareness and Perceptions

19. Where do you buy the vegetables you sell from?
    Farm gate ( )   Wholesale market ( )   Retail Market ( )   Other ( )
20. If farm gate, do you have any reason (s) why you buy from these farm gates?
    Yes ( )   No ( )
21. If yes, what are your reasons?
    List reasons..
22. Do you know of the source of water farmers use to irrigate their crops?
    Yes ( )   No ( )
23. If you know the source is drain water, would you still buy the vegetables?
    Yes ( )   No ( )
    List reasons for Yes or No answer..
24. Are you aware of any health risks associated with the consumption of vegetables that are irrigated with drain water?   Yes ( )   No ( )
25. If Yes, what health risks do you know of?
    List health risks...
26. Do you consume some of the vegetables yourself?   Yes ( )   No ( )
27. If you use the vegetables yourself, how many times in a week do you consume them uncooked (i.e. prepare it as salad)? [ ]

28. How much of the following vegetables do you use to prepare salad for one meal?

**Vegetable**	**Quantity used**
a) Lettuce	
b) Cabbage	
c) Spring Onion	
d) Carrot	

E. Personal Background

29. Sex: Male ( ) Female ( )

30. Age (yrs.): [ ]

31. Religion: Christian ( ) Moslem ( ) Traditional worshipper ( ) others ( )

32. Education: No formal education ( ) Primary education ( ) JHS/MS ( )
    SHS/A-level ( ) Polytechnic/University ( )

Date: **Time:**

Enumerator ID: **Location ID:**

Appendix H. Questionnaire for produce buyers (market)

QUESTIONNAIRE FOR PRODUCE BUYERS (MARKET)

A. Consumption Patterns

1. Which of these vegetables do you normally buy most at the market? Lettuce ( )
   Carrot ( ) Spring onion ( ) cabbage ( )
2. How often in a week do you buy lettuce/cabbage from the market? [ ]
3. What do you normally do at home to clean these vegetables?
   Wash with clean water ( ) Wash in salt water ( ) Add a disinfectant ( )
   Other ( ) Specify...
4. Mostly, do you consume these vegetables cooked or uncooked?
   Cooked ( ) Uncooked ( )
5. If vegetables are sometimes consumed uncooked, how often in a week is this done (i.e. consumed as salad)? [ ]
6. On average, how many of these vegetables do you use for one salad meal in a day?
   a). Lettuce [ ] b). Cabbage [ ] c). Spring Onion [ ] d). Carrot [ ]
7. How many people in the house consume the salad in a day? [ ]

B. Health Risk Awareness and Perceptions

8. Are you aware of the source of the vegetables you buy at the market? Yes ( ) No ( )
9. What is the main reason why you buy from this market vendor and not from others?
   Write down the main reason...
10. Do you think the source of water farmers use to irrigate the vegetables could have influence on your decision to buy them? Yes ( ) No ( )
11. Would you still buy these produce or not if you were aware that it was irrigated with wastewater (drain water)? Buy ( ) Not buy ( )
12. Are you aware of any health risks associated with the consumption of produce that are irrigated with wastewater? Yes ( ) No ( )
13. If Yes, what are the main health risks/disease you know of?
    Write down main health risks mentioned...
    ....
14. Have you had diarrhoea within 2 weeks after consuming salads before?
    Yes ( ) No ( ) cannot remember ( )

C. Environmental Conditions and Health Status

15. Are you generally satisfied with how produce are displayed for sale at the markets?
    Yes ( ) No ( )
16. Are you satisfied with the general environmental conditions at the produce vending site?
    Yes ( ) No ( )
17. If No, can you give reasons?...
    ....
18. What do you think can be done by market vendors to improve environmental sanitation at the market?
    Write down responses..
    ....

19. What do you think can be done by the government to improve environmental sanitation conditions at the market?
    Write down responses..

D. Personal Background

20. Sex: Male ( ) Female ( )
21. Age (yrs.): [ ]
22. Religion: Christian ( ) Moslem ( ) Traditional worshipper ( ) others ( )
23. Occupation: Government/Office worker ( ) Trading ( ) Vocational ( ) Other ( )

Enumerator ID: **Location ID:** **Date:**

Appendix I. Questionnaire for street food vendors

QUESTIONNAIRE FOR STREET FOOD VENDORS

A. Selling information

1. How many days in a week do you normally sell cooked rice/other food with salad? [ ]

2. On average, how much of these vegetables do you use to prepare salad for a day?
   a). Lettuce [ ] b). Cabbage [ ] c). Carrot [ ] d). Spring Onion [ ]

3. On average, how many customers buy rice with salad in a day? [ ]
4. How much time do you spend selling food at your vending site? [ ]

5. What do you normally do when less busy at your vending site?
   Chatting with friends ( ) Washing of utensils ( ) wait for customers ( )
   Other ( ) Specify...

B. Produce and Food Hygiene Practices

6. Where do you normally store the produce before using them for salad?
   At home ( ) At vending site ( ) use it immediately after buying ( )
   Other (Specify)
7. How do you store the produce? On the bare ground ( ) On a mat on the ground ( )
   In a box or container ( ) Other (Specify)
8. Where do you normally prepare the salad?
   At home ( ) At vending site ( ) Other ( ) Specify...
9. What do you normally do to clean the vegetables before salad preparation?
   Wash with clean water ( ) Wash in salt water ( ) Use a disinfectant (Vinegar).................. Other (Specify).................................
10. What is the source of water for washing the produce before salad preparation?
    Piped water ( ) well water ( ) Tanker services ( ) Other ( ).
11. Do you normally wash your hands before salad preparation? Yes ( ) No ( )
12. If Yes, what do you use to wash your hands? Only water ( ) Water and Soap ( ) Other ( )
13. What is your normal practice of serving the salad to consumers?
    Bare hands ( ) Hand covered with a polythene bag ( ) Spoon/ladle ( ) Other ( )

C. Environmental Hygiene conditions

14. Where do you normally defaecate when you are at work on the farm?
    Public toilet ( ) Neighbour's/friend's toilet ( ) In a polythene bag ( ) Open Defaecation ( )

D. Health Risk Awareness

15. Where do you normally buy the vegetables you use to prepare the salad from?
    Farm gate ( ) Wholesale market ( ) Retail Market ( )
16. If farm gate, do you have any reason (s) why you buy from these farm gates?
    Yes ( ) No ( )
17. If yes, what are your reasons?
    List reasons..
18. Are you aware of the source of water farmers use to irrigate their crops?

Yes ( )   No ( )

19. If you know the source is drain water, would you still buy these vegetables?
    Yes ( )   No ( )
20. If yes, could you provide some reasons..
    ....
21. If No, could you provide some reasons...
    ....
22. Are you aware of any health risks associated with the consumption of vegetable salads that are irrigated with wastewater?   Yes ( )   No ( )
23. If Yes, what health risks do you know of?
    List health risks...
24. Do you consume some of the salads yourself?   Yes ( )   No ( )
25. If yes, how often in a week do you consume them? [ ]

26. Have you had any diarrhoea disease within 2 weeks after consuming salad foods?
    Yes ( )   No ( )   cannot remember ( )

E. Personal Background

27. Sex   Male ( )   Female ( )
28. Age (yrs.) [ ]
29. Religion   Christian ( )   Moslem ( )   Traditional worshipper ( )   others ( )
30. Education: No formal education ( )   Primary education ( ) JHS/MS ( )   SHS/A-level ( )   Polytechnic/University ( )
31. Do you do any other work apart from street food vending?   Yes ( )   No ( )
32. If Yes, how much time do you spend on each of them?
    Street food vending..........................   Other jobs...

Enumerator ID   **Location ID**   **Date**

Appendix J. Questionnaire for street food consumers

QUESTIONNAIRE FOR STREET FOOD CONSUMERS

A. Consumption Patterns

1. Do you normally take the salad or only the rice? Yes, I take ( ) No, I do not take ( )
2. If No, do you have any reasons why you don't take the salad?
   Write down reason(s)..
   ....
3. If you take the salad, how often in a week do you normally consume street salad food ("check-check"/others)? [ ]

4. What makes you prefer to buy and consume street foods with salad? (tick all answers)
   Cheap ( ) Convenient ( ) I just like it ( ) Other (specify).................

B. Health Risk Awareness and Perceptions

5. Does anything influence your decision to buy the food from one seller and not the other?
   Write down the main reason...
   ....
6. Are you aware of the source of water used to irrigate the vegetables used for the salad?
   Yes ( ) No ( )
7. Would you still buy or take the salad if you were aware that the vegetables used to prepare it were irrigated with drain water? Buy ( ) Not buy ( )
8. Are you aware of any health risks associated with the consumption of salad prepared from vegetables that are irrigated with drain water? Yes ( ) No ( )
9. If Yes, what is the main health risk/disease you know of? Write down the main health risk/disease mentioned
   ....
   ....

C. Environmental Conditions and Health Status

10. Are you generally satisfied with how the salads are prepared?
    Yes ( ) No ( ) don't know how they are prepared ( ) Other ( )
11. Are you satisfied with the general environmental conditions at the food vending site?
    Yes ( ) No ( )
12. If No, what do you think can be done to improve the environmental sanitation conditions at the vending sites?...
    ....
13. Have you ever had diarrhoea disease within 2 weeks after consuming lettuce salads?
    Yes ( ) No ( ) cannot remember ( )

D. Personal Background

14. Sex: Male ( ) Female ( )
15. Age (yrs.): [ ]
16. Religion: Christian ( ) Moslem ( ) Traditional worshipper ( ) others ( )
17. Occupation: Government/Office worker ( ) Trading ( ) Vocational ( )
    Other (specify)

Enumerator ID **Location ID** **Date**

Appendix K. Observation guide for farm workers

Observation Guide/Behaviour Record for Farm Workers
For each activity, record the time for performing the activity and also complete the exposure parameters. Observe the farmer from 7 – 10am.

**Activity**	**Time period for activity**	**Exposures**	**Time period for Exposures**
1. Bed Preparation	Start time:<br>End time:	a) Hand in contact with soil : Yes ( ) No ( )<br>b) Feet in contact with soil: Yes ( ) No ( )<br>c) Hand contact with face/mouth: Yes ( ) No ( )	Hand contact time:<br>Feet contact time:<br>Tally for hand contact:
2. Transplanting	Start time:<br>End time:	a) Hand in contact with soil : Yes ( ) No ( )<br>b) Feet in contact with soil: Yes ( ) No ( )<br>c) Hand contact with face/mouth: Yes ( ) No ( )	Hand contact time:<br>Feet contact time:<br>Tally for hand contact
3. Soil tilling (Forking)	Start time:<br>End time:	a) Hand in contact with soil : Yes ( ) No ( )<br>b) Feet in contact with soil: Yes ( ) No ( )<br>c) Hand contact with face/mouth: Yes ( ) No ( )	Hand contact time:<br>Feet contact time:<br>Tally for hand contact:
4. Removal of weeds	Start time:<br>End time:	a) Hand in contact with soil : Yes ( ) No ( )<br>b) Feet in contact with soil: Yes ( ) No ( )<br>c) Hand contact with face/mouth: Yes ( ) No ( )	Hand contact time:<br>Feet contact time:<br>Tally for hand contact:
5. Watering (Irrigation) of crops (indicate the source of irrigation water, e.g. drain water)	Start time:<br>End time:	a) Hand in contact with soil : Yes ( ) No ( )<br>b) Feet in contact with soil: Yes ( ) No ( )<br>c) Hand in contact with irrigation water: Y/N<br>d) Feet in contact with irrigation water: Y/N	Hand contact time:<br>Feet contact time:<br>Hand contact time:<br>Hand contact time:

6. Mixing of chemicals (pesticides/insecticides) for spraying	Start time:<br>End time:	a) Farmer in hand gloves: Yes ( ) No ( )<br>b) Feet in contact with soil: Yes ( ) No ( )	
7. Spraying	Start time:<br>End time:	a) Farmer in nose mask : Yes ( ) No ( )<br>b) Farmer in hand gloves: Yes ( ) No ( )<br>c) Feet in contact with soil: Yes ( ) No ( )	Feet contact time:
8. Harvesting (including packing of produce into sacks)	Start time:<br>End time:	a) Hand in contact with soil : Yes ( ) No ( )<br>b) Feet in contact with soil: Yes ( ) No ( )	Hand contact time:<br>Feet contact time:
9. Transportation of harvested produce to road side	Start time:<br>End time:	a) Feet in contact with soil: Yes ( ) No ( )	Feet contact time:
10. Eating	Start time:<br>End time:	a) Farmer washed hands: Yes ( ) No ( )<br>b) Farmer washed hands with water and soap: Yes ( ) No ( )<br>c) Farmer washed hands with only water: Yes ( ) No ( )	
11. Farmer Idle (Indicate activities)	Start time:<br>End time:		

Enumerator ID: ***Location ID:*** ***Date:***

Appendix L. Observation guide for market vendors

Observation Guide – Behaviour Record (Market Vendors)
For each activity, record the time for performing the activity and also complete the exposure parameters. Observe the Vendor from 7 – 10am.

**Activity**	**Time period for activity**	**Exposures**	**Time period for Exposures**
1. Washing of carrots	Start time:<br>End time:	a) 'Silver' (wire) sponge used : Yes ( ) No ( )<br>b) Water used for washing dirty: Yes ( ) No ( )<br>c) Number of times water used for washing changed: ☐<br>d) Where wash water was disposed off: on bare ground ( ) drain ( ) Other (specify).............	e) Time period water used unchanged: ☐
2. Washing of Lettuce	Start time:<br>End time:	a) Water used for washing dirty: Yes ( ) No ( )<br>b) Number of times water used for washing changed: ☐<br>f) Where wash water was disposed off: on bare ground ( ) drain ( ) Other (specify).............	c) Time period water used unchanged: ☐
3. Cutting/removal of waste parts of cabbage	Start time:<br>End time:	a) Cabbage in contact with bare ground: Yes ( ) No ( )	c) Time period cabbage in contact with bare ground:

		b) Where cabbage displayed/stored after cleaning: bare ground ( ) table top ( ) basket ( ) other (specify)..........	
4. Cutting/removal of waste parts of spring onion	Start time:<br>End time:	a) Spring onion in contact with bare ground: Yes ( ) No ( )<br>b) Where spring onion displayed/stored after cleaning: bare ground ( ) table top ( ) basket ( ) other (specify)..........	c) Time period spring onion in contact with bare ground:
5. Unpacking of produce from sacks/polythene bags	Start time:<br>End time:	a) Where produce unpacked to: bare ground ( ) table top ( ) basket ( ) other (specify)..........	b) Time period produce in contact with bare ground:
6. Sweeping at vending site	Start time:<br>End time:	a) Rubbish collected immediately after sweeping: Yes/No<br>b) Where rubbish was kept in: Basket ( ) Polythene bag ( ) taking to skip container outside/within market ( ) Other ( )<br>c) Is rubbish collected covered if it is kept at vending site? Yes ( ) No ( )	d) Time period rubbish kept at vending site:<br>e) Time period rubbish kept at vending site uncovered:
7. Packing/displaying of produce for sale	Start time:<br>End time:	a) Where produce unpacked to: bare ground ( ) table top ( ) basket ( ) Other (specify)..........<br>b) Flies hovering on produce: Yes ( ) No ( )	

8. Sprinkling of water unto produce	Start time:<br>End time:	a) Type of water used: piped water ( ) well water ( ) Sachet water ( ) Other (specify)............<br>b) How water was sprinkled: hand ( ) Sachet water ( ) use of foam ( ) Other (specify)..........	
9. Eating	Start time:<br>End time:	a) Vendor washed hands: Yes ( ) No ( )<br>b) Vendor washed hands with water and soap: Yes ( ) No ( )<br>c) Type of water used to wash hands: Piped water ( ) well water ( ) Sachet water ( ) Other (specify)............	
10. Vendor Idle (Indicate activities)	Start time:<br>End time:		

Enumerator ID: ***Location ID:*** ***Date:***

Appendix M. Observation guide for street food vendors

Observation Guide – Behaviour Record (Street Food Vendors)
For each activity, record the time for performing the activity and also complete the exposure parameters. Observe the Vendor from 6 – 9 pm.

**Activity/Item**	**Time period for activity**	**Exposures**	**Time period for Exposures**
1. Preparation of Salad	Start time:<br>End time:	a) Vendor washed hands before handling salad: Yes ( ) No ( )<br>b) Source of water used to wash vegetables (ask vendor if not sure of the source) :Piped water ( )<br>Sachet water ( ) Well water ( ) Other (specify)......................<br>c) How vegetables were washed: Water only ( ) Salt water ( ) Vinegar ( ) Other (Specify).............	
2. Storage of salad		a) Type of receptacle salad is kept in before serving to customers (Specify) ☐<br>b) Is salad covered in receptacle? Yes ( ) No ( )	c) Time period salad was left uncovered: ☐
3. Serving of rice with salad	Start time:<br>End time:	a) Was salad covered before serving was done? Yes ( ) No ( )	d) Time period salad was left uncovered: ☐

		b) How salad is served to customers: Hand ( ) Spoon/Ladle ( ) Other (specify)...................<br>c) How chicken/meat/fish is served to customers: Hand ( ) Spoon/Ladle ( ) Other (Specify).................	
4. Washing of Plates	Start time:<br>End time:	a) Source of water used to wash plates: Piped water ( ) Sachet water ( ) Well water ( ) Other (specify)......................<br>b) Number of times water used for washing plates changed: ☐<br>c) Where wash water was disposed off: On bare ground ( ) drain ( ) Other (specify).............	d) Time period before water used to washed plates was changed: ☐
5. Eating	Start time:<br>End time:	a) Vendor washed hands: Yes ( ) No ( )<br>b) Vendor washed hands with: Water only ( ) Water and soap ( ) Other (specify).................<br>c) Type of water used to wash hands: Piped water ( ) well water ( ) Sachet water ( ) Other (specify)............	
6. Vendor Idle (Indicate activities)	Start time:<br>End time:		

7. Number of Customers	Tally the number of customers within the observation period		
8. Environmental Conditions at vending site		a) How refuse is managed/kept at vending site: In a covered receptacle ( ) In uncovered receptacle ( ) Left on the vending ground ( ) Other (Specify).................................<br>b) Vending site concreted: Yes ( ) No ( )<br>c) Floor of vending site swept/kept clean: Yes ( ) No ( )<br>d) Vending site within 3m of open drain: Yes ( ) No ( )<br>e) Flies at vending site: Yes ( ) No ( )	

Enumerator ID: ***Location ID:*** ***Date:***
